# Cardiac, reproductive, and brain toxicity of zinc oxide nanoparticles in male rats: Focus on morphological alterations and oxidative stress markers

**DOI:** 10.1016/j.bbrep.2025.102336

**Published:** 2025-10-31

**Authors:** Fatemeh Mirzaei, Amir Mirzaei, Sara Soleimani Asl

**Affiliations:** a Research Center for Molecular Medicine, Hamadan University of Medical Sciences, Hamadan, Iran; b Hazegh Medical Laboratory, Tehran, Iran; c Department of Anatomy, School of Medicine, Hamadan University of Medical Sciences, Hamadan, Iran

**Keywords:** Malondialdehyde, Oxidative stress, Zinc oxide nanoparticle, Rats

## Abstract

Our bodies are exposed to different types of nanoparticles in our daily routine. Hence, this experiment aimed to measure the effects of different doses of zinc oxide nanoparticles (ZnO NPs) on various organs in Wistar rats.

Wistar rats were randomly divided into 6 groups as follows: group 1: control, group 2: control rats +5 mg/kg ZnO NPs, group 3: control rats +10 mg/kg ZnO NPs, group 4: control rats +25 mg/kg ZnO NPs, group 5: control rats +50 mg/kg ZnO NPs, and group 6: control rats +100 mg/kg ZnO NPs. The ZnO NPs were administrated orally for one month, and then the heart, hippocampus, and testis were removed and used for more analysis. The malondialdehyde (MDA) levels, total oxidant status (TOS), and total antioxidant capacity (TAC) were measured. For histological analysis, the tissues were analyzed by hematoxylin-eosin staining.

TAC significantly decreased in the hippocampus, heart, and testis of rats treated with 10–100 mg/kg ZnO NPs compared to the control (P < 0.05). The TOS and MDA levels significantly increased by 25–100 mg/kg ZnO NPs compared to the control (P < 0.05). Histological findings show that the structure of the hippocampus, heart, and tissues in the rats received 5 was similar to the healthy control rats. While, 25–100 mg/kg ZnO NPs had harmful effects on these tissues.

Our findings show that high dosages of ZnO NPs induce oxidative stress and histopathological changes in the hippocampus, heart, and testis.

## Highlights

• High dosages of ZnO NPs increased oxidative stress in the heart, testis, and cortex.
• Low dosages of ZnO NPs reduce increased oxidative stress.
• High dosages of ZnO NPs disturb the structure of the heart, testis, and cortex.

## 1 Introduction

Nanoparticles (NPs) are described as particles with a size of 1–100 nm in at least one dimension. These particles have existed on Earth's system for billions of years and have a role in the emergence and evolution of organisms [1]. The use of nanoparticles is like a double-edged sword: on the one hand, they have toxic effects, and on the other hand, they have beneficial effects in medicine and industry [1].

Nanoparticles are used for enhanced imaging, cancer therapy, cancer diagnosis, drug delivery, and regenerative medicine. Some NPs have potential antimicrobial, antioxidant, anti-inflammatory, and cardioprotective effects. In this respect, zinc oxide NPs are the second most abundant metal oxide after iron. Zinc oxide nanoparticles (ZnO NPs) are safe and inexpensive, and they can be prepared easily. ZnO NPs are widely used in tissue engineering, implant coating, and wound healing. NPs are also used in the food industry to prevent spoilage and preserve colors via their antimicrobial activity. Zinc oxide NPs are used in LEDs, cosmetics, sunscreens, pigments, solar cells, food additives, and semiconductors. Exposure to zinc oxide NPs occurs via inhalation, intravenous, dermal contact, and oral (the main route). Wide use of zinc oxide NPs leads to human exposure unintentionally or intentionally [2,3].

Zinc is a divalent cation that is necessary for the activity of many enzymes in the body. This essential trace element regulates different physiological responses, including immune responses, differentiation, growth, development, and maturation [4]. Zinc also plays a role in the processes of transcription, homology, nucleic acid, and protein synthesis. Zinc has a crucial role in the structure of various proteins and many biochemical and metabolic processes such as metabolism, cellular respiration, and protection against free radicals [4,5]. About 10 % of the human body's proteins exert their role by binding with zinc and regulating different physiological responses. Furthermore, zinc maintains the function and structure of membranes and thus protects cells from oxidant agents in the host defense. Due to its antioxidant properties, zinc deficiency increases the rate of lipid peroxidation [6].

ZnO NPs contain a high amount of zinc and are easily absorbed from the digestive tract and ultimately enter various tissues. Although ZnO NPs have been shown to have high biocompatibility and some beneficial properties, there are some concerns about their toxic effects. ZnO NPs are widely used in industry and medicine. Hence, our body expose to it with different route, including inhalation, ingestion, skin penetration, and intravenous injection. Prolong use of ZnO NPs elevates the risk of ingestion exposure, potentially causing their accumulation in various tissues and inducing toxicity. Hence, it is essential to determine the potential toxicity effects of ZnO NPs, which have revealed pro-inflammatory and cytotoxic effects. Our previous experiments show that ZnO NPs has antioxidants and toxicity effects in animal models [7,8]. The harmful effects of ZnO NPs include hormonal disturbances, inflammation, DNA damage, membrane damage, apoptosis, and oxidative stress [9]. ZnO NPs can increase the production of reactive oxygen species (ROS) in different organs. These ROS can lead to oxidative stress, DNA damage, and consequently changes in cellular structure and functions. Previous studies showed that metal-based NPs can have toxic effects when administered orally, intraperitoneally, or intravenously. The vital tissues that absorb and interact with NPs include the brain, lungs, liver, spleen, and kidneys [10].

Previous studies have shown that high doses of NPs increase oxidative stress and reduce the total antioxidant capacity [11]. Furthermore, long-term use of nanoparticles can be associated with some pathological outcomes, including neurological disorders, nephrotoxicity and hepatotoxicity, splenic dysfunction, oxidative stress, apoptosis, and inflammation. Although the exact mechanism of damage caused by NPs is not yet fully understood, oxidative stress and inflammation seem to play an important role [12].

Some studies have shown that ZnO NPs have cytotoxic and proinflammatory effects and cause reactive oxygen species (ROS) generation in various tissues. The physicochemical properties of nanoparticles, including surface charge, particle size, and chemical composition, are the main factors in their ability to increase the production of reactive oxygen species (ROS), consequently leading to oxidative tissue damage [1]. The production of oxidative stress and inflammatory factors plays a central role in the pathology of many diseases [13]. However, other studies reported that ZnO NPs have potential antioxidant and anti-inflammatory effects [14]. In previous studies, we have investigated the effects of ZnO NPs on kidney, liver, and pancreas tissues, and our results indicate beneficial effects of low doses of ZnO NPs, while high doses had toxic effects [7,8]. We also evaluated the effects of ZnO NPs on non-alcoholic fatty liver disease (NAFLD)and diabetic animal models. The previous studies of the author showed that ZnO NPs protect the pancreas and liver from oxidative damage and normalized blood lipid, insulin, and glucose levels. [8,15]. Although the harmful and beneficial effects of some dosage of ZnO NPs were evaluated in different animal models, fewer studies have investigated the effects of various concentrations of ZnO NPs on oxidative stress and morphological changes in heart, brain, and testis tissues. In this experiment, the ZnO NPs were chosen at lower doses (5–10 mg/kg), mid dose (25 mg/kg), and higher doses (50–100 mg/kg) to cover a broad-spectrum of toxicity effects.

## 2 Methods

### 2.1 Animals and treatment

In this experimental study, the male Wistar rats (200–220 g) were purchased from the Hamadan University of Medical Sciences (Hamadan, Iran). Rats were adapted in animal houses for 14 days, at a temperature of 22 ± 2 °C, with 55 % humidity. Dark and light circles were maintained for 12 h each and animals had free access to water and a standard chow diet. After that, rats were randomly divided into 6 groups as follows (n = 6) based on previous experiments [7,8]: group 1: control rats received a chow diet, group 2: control rats received 5 mg/kg ZnO NPs, group 3: control rats received 10 mg/kg ZnO NPs, group 4: control rats received 25 mg/kg ZnO NPs, group 5:control rats received 50 mg/kg ZnO NPs, and group 6: control rats received 100 mg/kg ZnO NPs. The ZnO NPs (Nano Nanosany Co. Mashhad, Iran) were administrated orally for one month.

### 2.2 Sampling

The rats were anesthetized by isoflurane (2.5 %–5 %) after overnight fasting and the hippocampus, heart, and testis were removed and washed with phosphate buffer saline (PBS). A portion of tissues were fixed in 10 % formalin for histopathological examination, and the rest was frozen in liquid nitrogen and stored at −70 °C for evaluation of antioxidant factors.

### 2.3 Tissue homogenization

The hippocampus, heart, and testis of each rat were homogenized in PBS containing protease inhibitors and then centrifuged at 12000 rpm for 15 min, and the supernatant was collected and stored at −80 °C for antioxidant tests.

### 2.4 Total protein measurement

The protein content in the heart, testis, and hippocampus was measured by Bradford method using bovine serum albumin as a standard.

### 2.5 Total antioxidant capacity (TAC)

The TAC levels were measured based on CUPRAC method using the available kit (Kiazist, Iran). In this method, the reducing agents in the sample reduce the Cu^2+^ to Cu ^+^ producing color which is measured at 450 nm. The absorption is directly associated with the amounts of total antioxidants. Briefly, 30 μL of tissue homogenate was added to each well. Then 150 μL of TAC working (150 μL TAC buffer, 2 μL chromogen, and 2 μL CU^+2^ solution) solution was added to each well. The samples were incubated at room temperature for 1 h and the absorbance of the plate at a wavelength of 450 nm. The TAC levels are calculated based on the standard curve [16,17].

### 2.6 Total Oxidant Status (TOS)

TOS refers to the nitrogen-free species (RNS) and oxygen-free species (ROS) in the sample. The TOS levels were determined based on xylenol orange dye using the available kit (Kiazist, Iran). In this method, the oxidant compounds in the sample oxidized the ferrous ion to the ferric ion which produce color. Briefly, 50 μl of the sample was added to each well. 200 μl of the working solution was added to each well. The samples were incubated at room temperature for 20 min and then the absorbance was read at 560 nm [17].

### 2.7 Malondialdehyde (MDA)

The levels of MDA were measured by thiobarbituric acid assay using the available kit (Kiazist, Iran). In this experiment, MDA forms a complex with thiobarbituric acid, which absorbs at a wavelength of 532–560 nm. About 20–30 mg of tissue was washed with PBS buffer and mixed with 3 μl of butylated hydroxytoluene (BHT) and 300 μl of MDA-lysis buffer. The samples were homogenized and then centrifuged at 6000 g for 10 min. The supernatant was separated and used for MDA measurement. 200 μL of sample was added to each microtube and then 600 μL of thiobarbituric acid (TBA) solution was added to each tube. The microtubes were incubated for 1 h at 95 °C. Then they were cooled for 10 min at room temperature. Then 200 μL of the sample supernatant was transferred to the plate and its absorbance was read at a wavelength of 560 nm [17].

### 2.8 Antioxidant enzymes activity

The activity of antioxidant enzymes, including superoxide dismutase (SOD), glutathione peroxidase (GPX), and catalase (CAT) were measured in the heart, brain, and testis using colorimetric kits (Kiazist, Iran).

### 2.9 Histological evaluation

For histological analysis, the tissues were washed in PBS and placed in 10 % formalin. Following fixation, the samples were dehydrated, and a paraffin block was prepared. From each paraffin block, 10 μm sections were prepared and stained with hematoxylin-eosin (H& E). Several random non-overlapping fields were selected and analyzed. The histopathological alterations were examined by a light microscope (echo LAB CMOS 16004DSDW21) equipped with a Motic Moticam ^3+^ digital camera and analyzed by Image-J software.

### 2.10 Statistical analysis

Data analysis was performed using SPSS 16 software and results were reported as Mean ± SD. Tukey's post hoc test was done to compare the mean differences among various groups.

## 3 Results

### 3.1 Oxidative stress markers of the hippocampus

The ANOVA analysis shows significant changes in the TAC in the hippocampus of rats after treatment with ZnO NPs (P < 0.05). TAC was significantly reduced in the hippocampus of rats treated with 10, 25, 50, and 100 mg/kg ZnO NPs compared to the control (P < 0.05, P < 0.05, P < 0.001, and P < 0.001, respectively). However, TAC levels show a significant increase in animals treated with 5 mg/kg ZnO NPs compared to the control (P < 0.05)

The MDA levels showed a significant increase in the hippocampus of rats after treatment with 10, 25, 50, and 100 mg/kg ZnO NPs compared to the control (P < 0.001, P < 0.001, P < 0.001, and P < 0.001, respectively). The change of MDA in the hippocampus was not significant in animals treated with 5 mg/kg ZnO NPs compared to the control (P > 0.05).

**Fig. 1:**
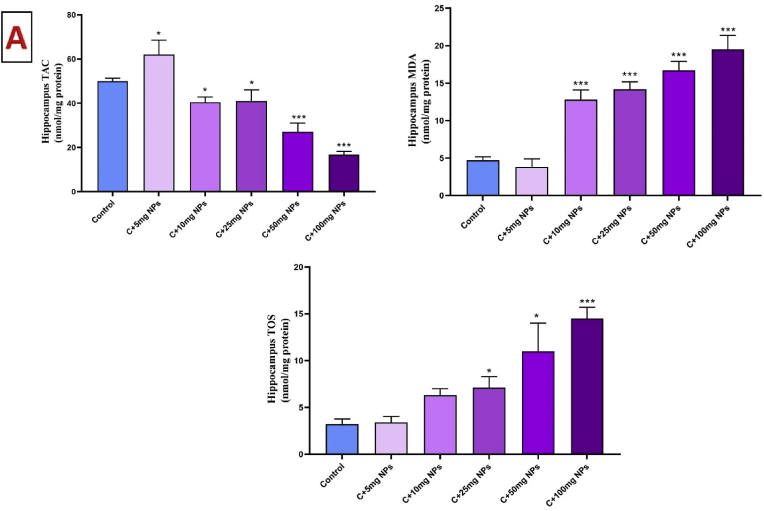

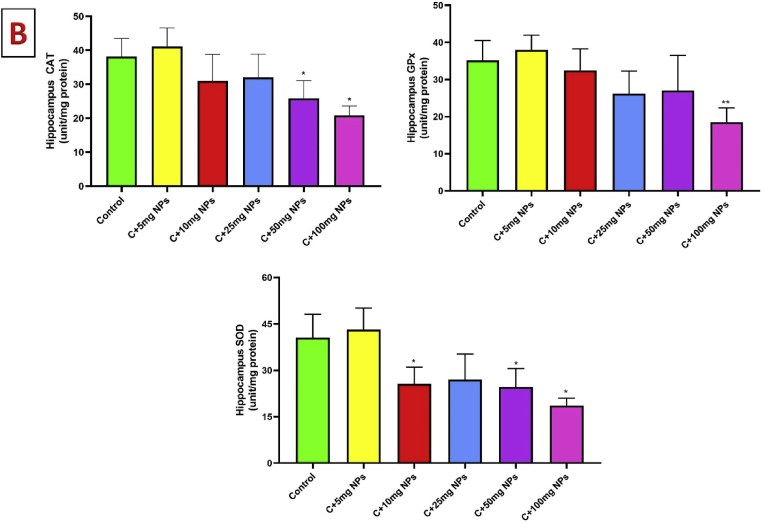
Effects of ZnO NPs on hippocampus oxidative stress markers (A) and antioxidant enzymes (B). Results are presented as a Mean ± SD. TOS: total oxidant status, TAC: total antioxidant capacity, MDA: malondialdehyde, CAT: catalase, GPX: glutathione peroxidase, SOD: superoxide dismutase. ∗P : < 0.05, ∗∗: p < 0.01, ∗∗∗: p < 0.001 compared to the control group.

Similarly, the TOS in the hippocampus showed a significant increase in rats after treatment with 25, 50, and 100 mg/kg ZnO NPs compared to the control (P < 0.05, P < 0.05, and P < 0.001, respectively). The levels of TOS showed no significant difference between animals treated with 5 and 10 mg/kg ZnO NPs compared to control rats (P > 0.05) (Fig. 1).

The activity of CAT significantly reduced in the hippocampus of male rats after treatment with 50, and 100 mg/kg ZnO NPs as compared to the control (P < 0.05). GPx activity only reduced by 100 mg/kg ZnO NPs as compared to the control (P < 0.001). SOD activity also reduced by 50 and 100 mg/kg ZnO NPs as compared to the control (P < 0.01 and P < 0.001, respectively), while increased by 5 mg/kg ZnO NPs as compared to the control (P < 0.05).

### 3.2 Histopathological changes of the hippocampus

**Fig. 2:**
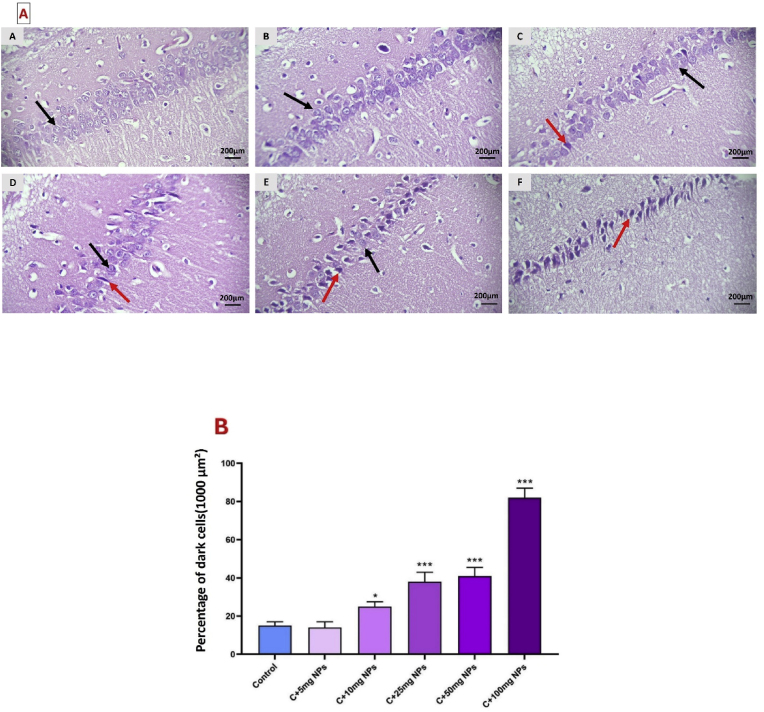
Light microscopic pictures of the CA1 region of the hippocampus as determined by hematoxylin and eosin (H & E) staining (A, original magnification 40 0×) and quantified as a percentage of dark cells (B). Values are presented as mean ± SD. ∗P : < 0.05, ∗∗: p < 0.01, ∗∗∗: p < 0.001 compared to the control group. A: Control, B: C + 5 mg/kg NPs, C: C+10 mg/kg NPs, D: C + 25 mg/kg NPs, E: C + 50 mg/kg NPs, F: C + 100 mg/kg NPs. Black arrow: intact neuron, red arrow: dark cells.

Histological findings show that the structure of the hippocampus in the groups receiving doses of 5 and 10 was like the healthy control group. While neuronal cell destruction, vacuolation with dystrophic changes in the form of small, pyknotic, and hyperchromatic nuclei was observed in the pyramidal cells in CA1 hippocampus were observed in the group receiving 25–100 mg/kg ZnO NPs. Dilation of blood vessels, abnormal Nissl granule distribution, and chromatolysis changes were not observed in treated animals. However, the highest concentration of ZnO NPs caused neuron necrosis, vacuolation, shrunken hyperchromatic nuclei, and accumulation of microglia cells around blood vessels (Fig. 2).

### 3.3 Oxidative stress markers of heart tissue

TAC significantly decreased in the heart of male rats after treatment with high dosages, but a significant increase was observed in rats that received 5 mg/kg ZnO NPs (P < 0.05). Treatment of rats with 10, 25, 50, and 100 mg/kg ZnO NPs significantly reduced TAC compared to the control (P < 0.05, P < 0.001, P < 0.001, and P < 0.001, respectively).

Statistical analysis showed that the levels of MDA in the heart tissue were reduced by 5 mg/kg concentrations of ZnO NPs (P < 0.05). MDA significantly increased in the heart of male rats after treatment with 10, 25, 50, and 100 mg/kg ZnO NPs compared to the control (P < 0.001,).

**Fig. 3:**
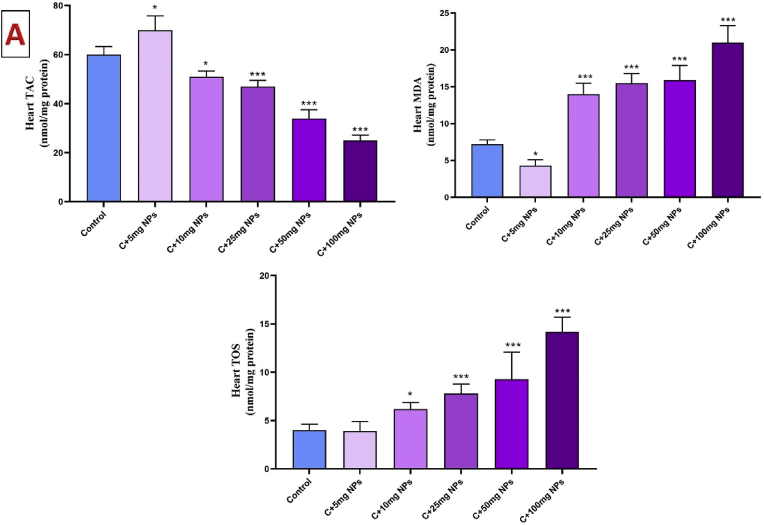

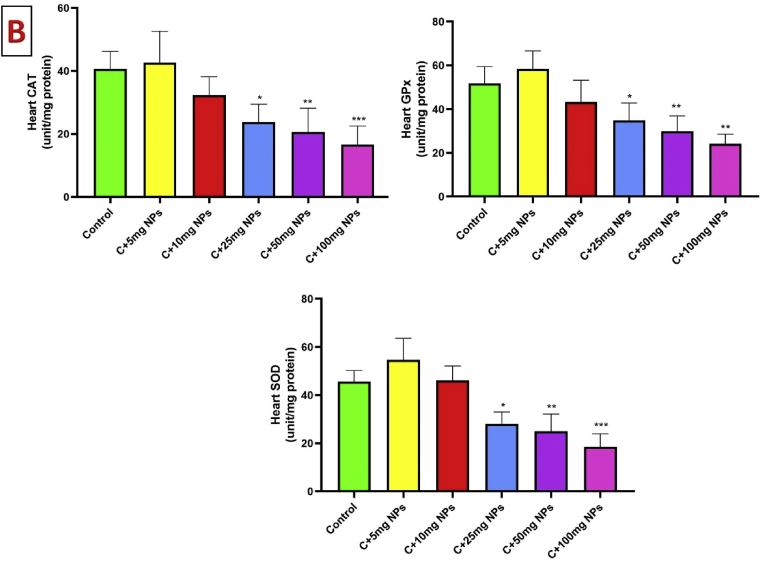
Effects of ZnO NPs on heart oxidative stress markers (A) and antioxidant enzymes (B). Results are presented as a Mean ± SD. TOS: total oxidant status, TAC: total antioxidant capacity, MDA: malondialdehyde, CAT: catalase, GPX: glutathione peroxidase, SOD: superoxide dismutase. ∗P : < 0.05, ∗∗: p < 0.01, ∗∗∗: p < 0.001 compared to the control group.

Statistical analysis revealed a significant increase in TOS levels in the heart of treated animals. When rats treated with ZnO NPs at the concentration of 10, 25, 50, and 100 mg/kg showed a significant increase in TOS levels compared to the control (P < 0.05, P < 0.001, P < 0.001, and P < 0.001, respectively). The change of TOS in the heart was not significant in animals treated with 5 mg/kg ZnO NPs compared to the control (P > 0.05) (Fig. 3).

The activity of CAT significantly reduced in the heart of male rats after treatment with 25, 50, and 100 mg/kg ZnO NPs as compared to the control (P < 0.05, P < 0.01, and P < 0.001, respectively). GPx activity also reduced 25, 50, and 100 mg/kg ZnO NPs as compared to the control (P < 0.05, P < 0.01, and P < 0.01, respectively). SOD activity also reduced by 25, 50 and 100 mg/kg ZnO NPs as compared to the control (P < 0.05, P < 0.001 and P < 0.001, respectively).

### 3.4 Histopathological changes of the heart

**Fig. 4:**
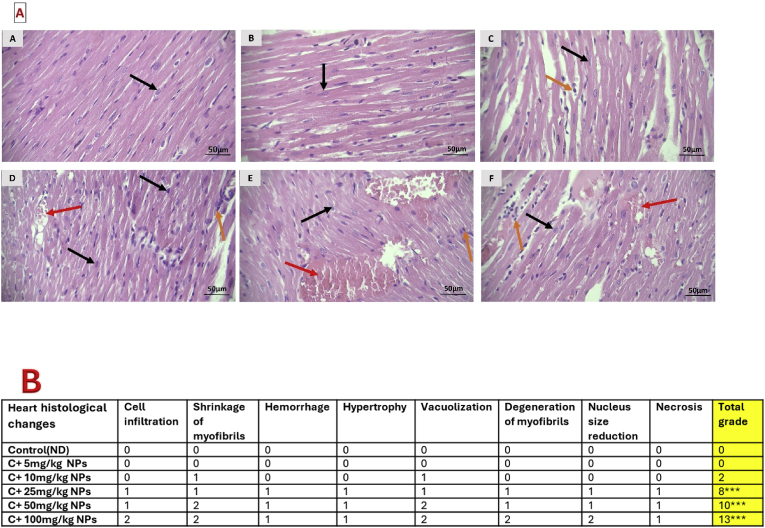
Light microscopic pictures of the heart stained by hematoxylin and eosin (H & E) (**A**, original magnification 40 × ) and quantified (**B**). Values are presented as mean ± SD. ∗P : < 0.05, ∗∗P < 0.01 and ∗∗∗P < 0.001 as compared to the control or normal diet (ND) group (n = 4). A: Control, B: C + 5 mg/kg NPs, C: C+10 mg/kg NPs, D: C + 25 mg/kg NPs, E: C + 50 mg/kg NPs, F: C + 100 mg/kg NPs. 0 = No damage,1 = Mild injury, 2 = Moderate injury, 3 = Severe injury. Black arrow: nucleus of the cardiac cell, red arrow: hemorrhage, orange arrow: infiltration of mononuclear cells.

Histopathological analysis showed that the cardiac muscle cells of the control animals were normal in morphology with an euchromatin oval nucleus, while sarcoplasm displayed a cross-striation sarcomere along with transparent intercalated discs. However, ZnO NPs at the dosage of 25–100 mg/kg led to the disorganization of myofibrils, along with some acidification of the sarcoplasm, congestion, severe extra-cellular hemorrhage, vacuolation, and leukocyte infiltration, and moderate hypertrophy. Treatment with low dosages, especially 5 and 10 mg/kg ZnO NPs revealed nearly normal heart structure (Fig. 4).

### 3.5 Oxidative stress markers of testis

**Fig. 5:**
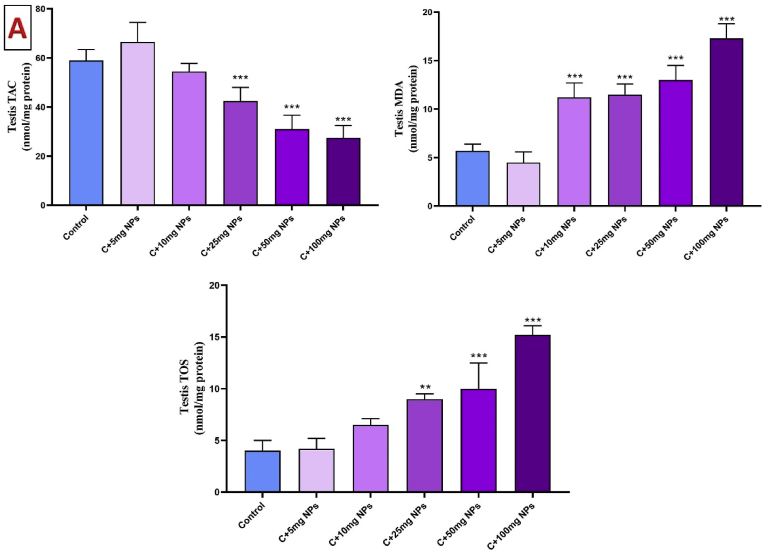

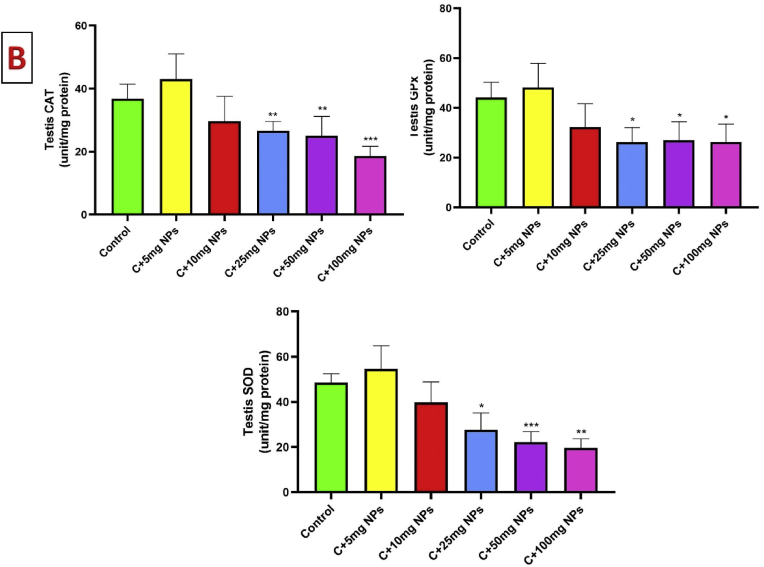
Effects of ZnO NPs on testis oxidative stress markers (A) and antioxidant enzymes (B). Results are presented as a Mean ± SD. TOS: total oxidant status, TAC: total antioxidant capacity, MDA: malondialdehyde, CAT: catalase, GPX: glutathione peroxidase, SOD: superoxide dismutase. ∗P : < 0.05, ∗∗: p < 0.01, ∗∗∗: p < 0.001 compared to the control group.

The results of oxidative markers in the testis revealed significant changes in different treated groups (P < 0.05). As seen in Fig. 5, the administration of ZnO NPs at the dosage of 5, and 10 mg/kg had no toxicity effect. However, ZnO NPs at the dosage of 25, 50, and 100 mg/kg significantly reduced the TAC in the testis of rats compared to the control (P < 0.001, P < 0.001, and P < 0.001, respectively).

Treatment of rats with ZnO NPs at the dosage of 10, 25, 50, and 100 mg/kg showed a considerable increase in the MDA in the testis of rats when compared to control animals (P < 0.001, P < 0.001, and P < 0.001, respectively). No significant differences in MDA levels were observed in the animals treated with 5 mg/kg ZnO NPs when compared to the control (P > 0.05).

The TOS was significantly higher in animals treated with high dosages of ZnO NPs. When animals treated with ZnO NPs at the dosage of 25, 50, and 100 mg/kg, showed a significant increase in testis TOS levels compared to the control (P < 0.01, P < 0.001, and P < 0.001, respectively). However, ZnO NPs at the dosage of 5 and 10 mg/kg showed a non-significant change compared to the control (Fig. 5).

The activity of CAT significantly reduced in the testis of male rats after treatment with 25, 50, and 100 mg/kg ZnO NPs as compared to the control (P < 0.01, P < 0.01, and P < 0.001, respectively). GPx activity also reduced 25, 50, and 100 mg/kg ZnO NPs as compared to the control (P < 0.05). SOD activity also reduced by 25, 50 and 100 mg/kg ZnO NPs as compared to the control (P < 0.05, P < 0.001 and P < 0.01, respectively).

### 3.6 Histopathological changes of the testis

**Fig. 6:**
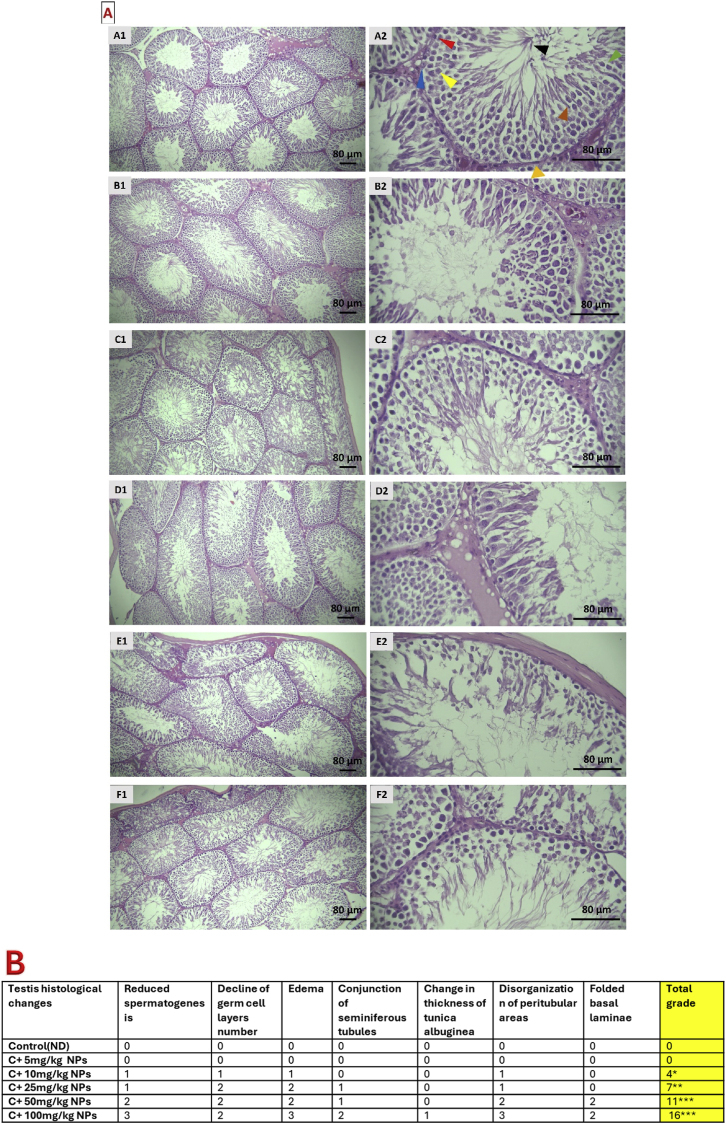
Light microscopic pictures of testis stained with hematoxylin and eosin (H & E), original magnification 40 × in the left column and 400 × in the right column (**A**) and quantified (**B**). Values are presented as mean ± SD. ∗P: < 0.05, ∗∗P < 0.01 and ∗∗∗P < 0.001 as compared to control group. A1, A2: Control, B1, B2: C + 5 mg/kg NPs, C1, C2: C+ 10 mg/kg NPs, D1, D2: C + 25 mg/kg NPs, E1, E2: C + 50 mg/kg NPs, F1, F2: C + 100 mg/kg NPs. 0 = No damage, 1 = Mild injury, 2 = Moderate injury, 3 = Severe injury. Red triangle: spermatogonia A, blue triangle: Spermatogonia B, yellow triangle: primary spermatocyte, green triangle: early spermatid, brown triangle: late spermatid, black triangle: spermatozoa, orange triangle: myoid cells.

Microscopic analysis showed that the testis of normal rats had normal histological structures, including packed seminiferous tubules, tunica albuginea, and interstitial tissue. The groups receiving ZnO NPs at the dosage of 25–100 mg/kg, showed irregular shape of tubules and a reduction in thickness of the germinal epithelium. These groups also showed mild congestion of interstitial tissue, increased tubule wrinkling, irregularity in the basement membrane, increased interstitial space, as well as a decrease in Leydig cells. These changes were more severe in high dosages. Hence, severe congestion of interstitial tissue, interstitial edema, testicular degeneration, and compaction of some seminiferous tubules were observed. Furthermore, infiltration of lymphatic cells in the space between the tubules was observed in high dosages of ZnO NPs. (Fig. 6).

## 4 Discussion

Zinc deficiency can lead to various disorders, including platelet aggregation, abnormal glucose levels, skin disorders, growth retardation, reproductive disorders, and immune dysfunction, which highlight the significant role of zinc in maintaining vital physiological activities [6]. The human body is exposed to different types of NPs because of nanotechnology development. NPs can be produced during natural processes such as weathering and mineral formation. Industrial emissions, forest fires, agricultural activities, and transportation produce huge amounts of NPs in the air. Therefore, the accumulation of NPs in the environment increases day by day. However, human exposure to nanoparticles can induce inflammation, oxidative stress, genetic damage, and inhibit cell division. In this respect, there is a potential risk of toxicity from their exposure to humans [1].

Human exposure to NPs during their processing, using food products, NPs production, and recycling. These particles can enter our body via different routes, such as oral ingestion, skin contact, and inhalation, leading to various toxic effects. The gastrointestinal tract is the main route of NPs entry into the human body. NPs enter the body directly through consumption or indirectly through the dissolution of nanoparticles in various compounds [18]. This nanoparticle has been used in food packaging and nutritional supplements due to its antibacterial properties and exceptional nutritive function. Interaction of NPs with the gastrointestinal tract occurs primarily through the digestion of a portion of cosmetics, food packaging materials, water, etc. It has been reported that NPs after absorption, are transferred to the bloodstream, and large amounts are introduced into the blood, liver, intestine, brain, kidney, lung, heart, and spleen. Once ZnO NPs enter the blood, they are rapidly distributed to various organs. Thus, evaluation of the toxicity effects and mechanisms of action can help us to know the effects of NPs on various organs which can guide the safe use of NPs [18]. However, the high use of ZnO NPs increases the toxicity risks. Thus, evaluation of the effects of ZnO NPs on normal animals can help us to know the toxicity and mechanism of this nanoparticle [19].

The results of this study showed that ZnO NPs at concentrations of 10–100 mg/kg changed the oxidative stress markers in the heart, testis, and brain tissues. In this study, ZnO NPs at doses of 25–100 mg/kg significantly increased TOS and MDA, while reducing TAC and GSH, and these doses caused damage to heart, testis, and brain tissue. Furthermore, ZnO NPs at doses of 25–100 mg/kg reduce the activity of SOD, GPx, and CAT. While the dose of 5 mg/kg increased TAC and GSH and reduced MDA and TOS. In the present study, ZnO NPs caused a dose-dependent increase in oxidative stress markers. Furthermore, high concentrations of ZnO NPs caused tissue damage in mentioned tissues. Hussein et al., reported that ZnO NPs reduce steroidogenesis in mice [20]. while, Bara et al., [21] showed that ZnO NPs increase steroidogenesis in TM-3 cells in vitro. Our previous study also showed that ZnO NPs at high dosages (50 and 100 mg/kg) induced oxidative stress, and inflammation and led to tissue damage. Pati et al. reported that treatment of rats with ZnO NPs (500 mg/kg body weight) caused DNA damage in peripheral blood cells and bone marrow. In another experiment, Abdulmalek showed that ZnO NPs (10 mg/kg) in diabetic rats increased memory learning and performance, improved redox status, and inflammation. ZnO NPs also reduced Bax and improved Bcl2 expressions in the hippocampus, indicating reduced apoptosis in the brain [22].

In the body the lysosomes uptake ZnO NPs and in the acidic pH induce their dissolution and release high concentration of Zn^2+^ ions. Free Zn^2+^ ions are toxic to many aquatic organisms. Hence, ZnO NPs at high concentration led to overload of Zn^2+^ ions, which can interrupt the zinc homeostasis, increase the dysfunction of mitochondria and damage the lysosomes, eventually lead to cell death. Furthermore, ZnO NPs at high concentration can accumulate in cell membrane and induce mechanical damage, affect physiological processes and cell signaling, and increase ROS production. ZnO NPs when are in small size can enter the nucleus and trigger DNA damage and ROS generation [23]. When NPs enter the cell, free zinc ions (Zn^2+^) are released from NPs, leading to mitochondrial damage, electron leakage in the electron transport chain, and ultimately inducing the production of ROS. Excessive production of ROS causes cellular damage and induces lipid peroxidation, and consequently cell damage and cell death. Furthermore, excessive increase in free Zn^2+^ levels causes damage to lysosomes, which results in the release of their contents into the cytoplasm and induces cell apoptosis and necrosis [24]. In addition to the above mechanisms, several factors can affect the toxicity of ZnO NPs, including animal species, route of administration, frequency of administration, and size and concentration of NPs [18].

ROS production by ZnO NPs causes DNA damage, protein degradation, and lipid peroxidation, ultimately causing cell membrane damage and an increase in harmful substances such as MDA. The MDA examination is a useful technique for examining increased lipid peroxidation due to its simplicity. In this study, animals that received ZnO NPs higher than 25 mg/kg showed a substantial rise in MDA levels [25]. In line with the present study, Xiao et al., [26] showed that zinc nanoparticles at high concentrations greatly increase lipid peroxidation. Lipid peroxidation is an important indicator of cellular damage and is measured by examining the concentration of MDA. The significant increase in MDA concentration by ZnO NPs plays a role in tissue dysfunction.

In this experiment, ZnO NPs increase ROS in the testis. Previous experiments have reported that NPs can negatively affect the function and structure of the reproductive system. The exact mechanisms are not completely understood. However, NPs can induce oxidative stress, inflammation, and DNA damage, which induce cell injury and infertility. Some experiments have established the susceptibility of testis to different types of NPs. ZnO NPs can cross the blood-testis barrier and accumulate in the reproductive system. The accumulation of ZnO NPs in this organ can affect spermatogenesis, causing disruptions in reproductive hormones and testicular structure. Hong et al., [27]showed that administration of ZnO NPs at the concentration 350 mg/kg ZnO NPs reduces sperm motility and hormone levels in Wistar rats. Pinho et al., also showed that ZnO NPs can cross blood-testis barrier and accumulate in the testis, leading to sex hormone disturbance, induce inflammation, oxidative stress, and germ cell damage [28]. Daoud et al., showed that administration of ZnO NPs (10 and 30 mg/kg) normalized serum testosterone, sperm count, decreased glutathione levels, and increased MDA in the testis of rats. They also showed that ZnO NPs increased the expression of steroidogenesis-related enzymes, including spermatocytes and most Sertoli cells (Star), and 3β-hydroxysteroid dehydrogenase (3β-HSD), cholesterol side-chain cleavage enzyme (CYP11A1) [29].

The results of this experiment also showed that a high concentration of ZnO NPs induced oxidative stress in the heart and brain. The brain is more susceptible to the toxicity effects of metallic NPs. The NPs through various mechanisms including apoptosis, oxidative stress, inflammation, autophagy, and disruption of sensory-motor signaling cascades can induce neurotoxicity. The brain consumes high levels of oxygen, high levels of iron, and has high levels of lipids, hence it is very prone to oxidative damage. It has been established that NPs can cross the blood-brain barrier and accumulate in the brain. Because of their nano size, NPs can cross the tight junctions of the brain and BBB [10]. ZnO NPs at a dosage of 5 mg/kg showed non-toxic or antioxidant activity in some cases. The anxiolytic properties of this nanoparticle ZnO NPs at a concentration of 5 mg/kg may be attributed to zinc function. Goma et al., show that ZnO NPs at the dosage of 5 mg/kg have positive effects on brain behavior that were evaluated by open field test, elevated plus maze, and Morris water maze tests. They also showed that this dosage reduces interleukin-6 (IL-6), tumor necrosis factor (TNF-α), and ROS levels, and normalizes antioxidant enzyme activities. However, they showed that high concentration had detrimental effects [30]. Our finding showed that a high concentration of ZnO NP leads to potential morphological changes in the heart, testis, and brain. The histopathological changes observed in various tissues were parallel to oxidative stress markers and previously published papers.

### 4.1 Study limitations

In this experiment the ZnO NPs administrated for only one month. However, it may not reflect the long-term effects in real-life. We also were not determined the exact signaling pathways. Another limitation that the inflammatory signaling pathways were not determined.

## 5 Conclusion

Our result shows that ZnO NPs at high dosages (25, 50, 100 mg/kg) induce oxidative stress and histopathological changes in the hippocampus, heart, and testis, while low doses increase total antioxidant capacity. However, ZnO NPs at low dosages (5 and 10 mg/kg) were not toxic and 5 mg/kg had beneficial effect on oxidative stress markers.

## Ethical approval

All parts of this experiment were approved by the Animals Ethics Committee of Hamadan University of Medical Sciences (Ethic code: IR.UMSHA.REC.1400.457). All experiments were conducted in accordance with the ARRIVE guidelines (Animal Research: Reporting of In Vivo Experiments) and adhered to the Guidance on the operation of the Animals (Scientific Procedures) Act 1986, as well as the EU Directive 2010/63 for the protection of animals used for scientific purposes.

## Funding

This study was supported by 10.13039/501100004697Hamadan University of Medical Sciences (Grant No. 140007205816).

## Declaration of competing interest

The authors declare the following financial interests/personal relationships which may be considered as potential competing interests: Fatemeh Mirzaei reports financial support was provided by Hamadan University of Medical Sciences, Hamadan, Iran. FATEMEH MIRZAEI has patent pending to NONE. If there are other authors, they declare that they have no known competing financial interests or personal relationships that could have appeared to influence the work reported in this paper.

## Data Availability

Data will be made available on request.
